# Hepatocyte Growth Factor Mediates Enhanced Wound Healing Responses and Resistance to Transforming Growth Factor-β_1_-Driven Myofibroblast Differentiation in Oral Mucosal Fibroblasts

**DOI:** 10.3390/ijms18091843

**Published:** 2017-08-24

**Authors:** Jordanna Dally, Jabur S. Khan, Alex Voisey, Chrisandrea Charalambous, Hannah L. John, Emma L. Woods, Robert Steadman, Ryan Moseley, Adam C. Midgley

**Affiliations:** 1 Stem Cells, Wound Repair & Regeneration, Oral & Biomedical Sciences, School of Dentistry, Cardiff University, Cardiff CF14 4XY, UK; dallyj@cardiff.ac.uk (J.D.); khanj5@cardiff.ac.uk (J.S.K.); voiseya@cardiff.ac.uk (A.V.); charalambousc@cardiff.ac.uk (C.C.); johnhl@cardiff.ac.uk (H.L.J.); woodse1@cardiff.ac.uk (E.L.W.); moseleyr@cardiff.ac.uk (R.M.); 2 Cardiff Institute of Tissue Engineering & Repair (CITER), Cardiff University, Cardiff CF10 3AX, UK; steadmanr@cardiff.ac.uk; 3 Wales Kidney Research Unit (WKRU), Systems Immunity Research Institute, Division of Infection and Immunity, College of Biomedical & Life Sciences, Cardiff University, Cardiff CF14 4XN, UK

**Keywords:** hepatocyte growth factor, oral mucosal fibroblasts, transforming growth factor-β_1_, proliferation, migration, differentiation

## Abstract

Oral mucosal wounds are characterized by rapid healing with minimal scarring, partly attributable to the “enhanced” wound healing properties of oral mucosal fibroblasts (OMFs). Hepatocyte growth factor (HGF) is a pleiotropic growth factor, with potential key roles in accelerating healing and preventing fibrosis. HGF can exist as full-length or truncated (HGF-NK), NK1 and NK2 isoforms. As OMFs display elevated HGF expression compared to dermal fibroblasts (DFs), this study investigated the extent to which HGF mediates the preferential cellular functions of OMFs, and the influence of pro-fibrotic, transforming growth factor-β_1_ (TGF-β_1_) on these responses. Knockdown of HGF expression in OMFs by short-interfering RNA (siHGF) significantly inhibited OMF proliferative and migratory responses. Supplementation with exogenous TGF-β_1_ also significantly inhibited proliferation and migration, concomitant with significantly down-regulated HGF expression. In addition, knockdown abrogated OMF resistance to TGF-β_1_-driven myofibroblast differentiation, as evidenced by increased α-smooth muscle actin (α-SMA) expression, F-actin reorganisation, and stress fibre formation. Responses were unaffected in siHGF-transfected DFs. OMFs expressed significantly higher full-length HGF and NK1 levels compared to patient-matched DFs, whilst NK2 expression was similar in both OMFs and DFs. Furthermore, NK2 was preferentially expressed over NK1 in DFs. TGF-β_1_ supplementation significantly down-regulated full-length HGF and NK1 expression by OMFs, while NK2 was less affected. This study demonstrates the importance of HGF in mediating “enhanced” OMF cellular function. We also propose that full-length HGF and HGF-NK1 convey desirable wound healing properties, whilst fibroblasts preferentially expressing more HGF-NK2 readily undergo TGF-β_1_-driven differentiation into myofibroblasts.

## 1. Introduction

Wound healing is a complex and highly ordered chain of events that leads to tissue repair. Although oral mucosal and dermal wounds proceed through similar stages of healing, oral mucosal wounds, in common with early-gestational foetal wounds [1], are characterized by minimal inflammatory and angiogenic responses, rapid healing/remodelling, and minimal scar formation. This is in contrast to adult dermal wounds, which are usually accompanied by prominent scar formation [2,3].

Oral mucosal healing is a consequence of phenotypic differences between the intra- and extra-oral wound cell populations. Oral mucosal fibroblasts (OMFs) have a distinctly increased ability to proliferate, migrate, and repopulate wounds, due to their enhanced proliferative lifespans [4,5,6]. OMF wound repopulation properties are further enhanced by their increased extracellular matrix (ECM) reorganisation [7,8,9,10,11]. More recently, proteomics profiling has demonstrated a marked difference in the production of a broad range of proteins between oral mucosal and vocal-fold fibroblasts [12], highlighting the importance of comparisons between cell populations isolated from different tissues. For example, OMFs also exhibit lower transforming growth factor-β_1_ (TGF-β_1_) expression, compared to their dermal fibroblast (DF) counterparts, and OMFs demonstrate resistance to TGF-β_1_-driven fibroblast-myofibroblast differentiation, thereby retaining their “non-scarring” phenotype [9,10,13,14]. The different responses to exogenous TGF-β_1_ treatment in DFs and OMFs are thought to be a consequence of differential Smad3 and hyaluronan regulation [15].

Furthermore, microarrays performed to elucidate the pathways underlying the preferential healing responses of OMFs have revealed numerous differentially expressed genes between patient-matched OMFs and DFs [6,16,17]. One of the genes identified and expressed at significantly higher levels in OMFs compared to DFs, was hepatocyte growth factor (HGF) [9,18,19,20].

HGF is a pleiotropic growth factor, and exhibits potent mitogenic, motogenic, morphogenic, angiogenic, anti-inflammatory, and anti-fibrotic properties in numerous cell types. These actions are mediated via the binding and activation of its receptor, tyrosine-protein kinase Met (c-MET) [21,22,23]. Consequently, HGF possesses multiple roles in normal organ/tissue development during embryogenesis, and in promoting tissue homeostasis, repair, and regeneration in numerous tissues, including skin and oral mucosa [3,21]. However, since HGF has also been implicated in the promotion of migration and angiogenesis, supporting cancer cell survival and metastasis [24,25], there is importance in clarifying the expression levels of HGF, its isoforms, and the roles they play, across different tissues. HGF can exist as a full-length, multi-domain protein, or as truncated peptide isoforms (HGF-NK), formed as a consequence of alternative splicing [26,27,28]. Full-length HGF is formed as an inert precursor (pro-HGF, 90 kDa), which is secreted and cleaved extracellularly at Arg494-Val495 into the biologically active form, by tightly regulated serine proteases: HGF activator, and matriptases [21,29].

The active form of HGF is a heterodimeric glycosylated protein, comprised of α- (55–60 kDa) and β- (32–34 kDa) chains, linked via a disulphide bridge [21,22]. The α-chain contains an N-terminal hairpin loop domain, and 4 kringle domains (NK1–NK4), which enable HGF receptor binding and activation. The β-chain has structural similarities to serine protease domains, however, lacks proteolytic activity, and contains a secondary receptor-binding site [21,22,30,31]. The truncated HGF isoform, NK1, contains the N-terminal hairpin and first kringle domain, whilst the NK2 variant also contains the second kringle domain [26,27,32]. NK1 is regarded as an agonist of the c-MET receptor, whilst NK2 is defined as a partial c-MET antagonist [28].

HGF binding to c-MET induces conformational changes through auto-phosphorylation of the cytoplasmic region, receptor homo-dimerisation and initiation of Grb2/Gab1 recruitment. This leads to the paracrine and autocrine activation of many intracellular signalling pathways, including extraceullar regulated kinases (ERK1/ERK2), phosphoinositide 3-kinase/protein kinase B (PI3K/Akt), signal transducer and activator of transcription 3 (STAT3), protein kinase C (PKC), and small GTP-binding proteins (Rac/Rho/Rap1) [21,22,28]. Such events result in a variety of overlapping signalling pathways being activated, and cellular responses induced.

The high levels of HGF expression may convey the “enhanced” proliferative, migratory, and differentiation resistance phenotype observed in OMF cultures, however, the extent to which full-length HGF and the NK1/NK2 isoforms mediate these OMF wound healing responses is currently unknown. This study assessed the effect of targeted HGF knockdown on OMF function, imperative for beneficial healing. In addition, these findings were compared to TGF-β_1_ treatments. To determine the anti-fibrotic and protective effects of HGF, knockdown and TGF-β_1_ were used in combination, to assess whether OMFs could be forced down a differentiation path to become myofibroblasts. Furthermore, full-length HGF and the NK1/NK2 isoforms were assessed under knockdown and TGF-β_1_ conditions, to elucidate their potential roles in OMF or DF function.

## 2. Results

### 2.1. Hepatocyte Growth Factor (HGF) Knockdown Inhibits Oral Mucosal Fibroblast Proliferation and In Vitro Scratch Wound Repopulation

**Figure 1:**
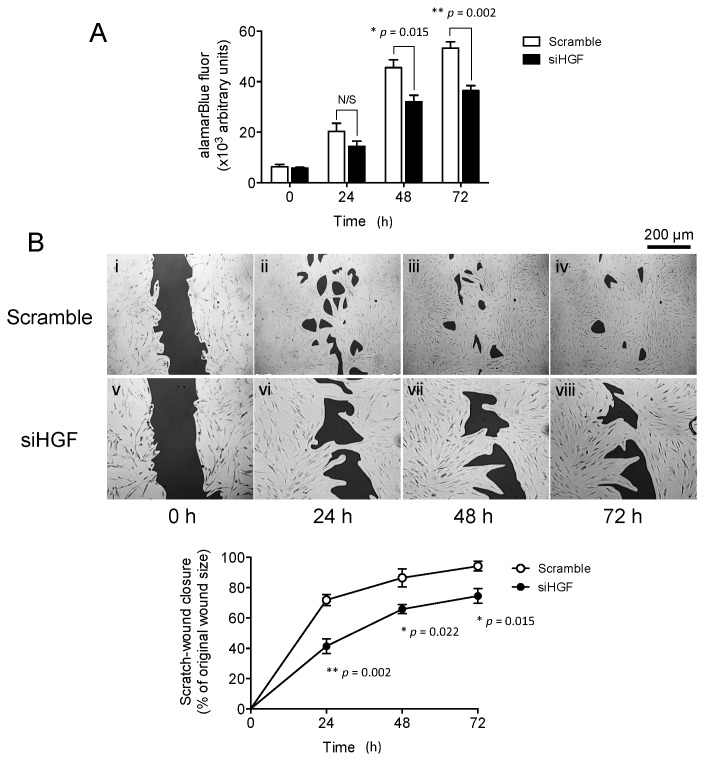
Hepatocyte growth factor (HGF) knockdown is detrimental to oral mucosal fibroblast (OMF) cell function. siHGF (solid bars/lines)- and scrambled siRNA (open bars/lines)-transfected, OMFs were assessed for effects on (**A**) proliferation and (**B**) in vitro scratch wound repopulation. Time-lapse images are representative of 3 independent scratch wound experiments (scale bar = 200 μm, original magnification 100×). Data is shown as mean ± standard error (S.E.) for *n* = 3 independent experiments (* *p* ≤ 0.05, ** *p* ≤ 0.01, vs. serum-free F-SCM controls; N/S, no significance).

We first sought to determine whether elevated HGF [18] was the primary source of “enhanced” cellular functions, and therefore, healing capabilities, in OMFs. Transfection of OMFs with siHGF significantly inhibited proliferation over 72 h in culture (Figure 1A). Proliferation was significantly inhibited in siHGF-transfected OMFs at 48 and 72 h (*p* = 0.015 and *p* = 0.002, respectively), compared to scrambled siRNA-transfected OMFs. This was maintained over 72 h in culture, with wound repopulation/closure significantly impaired by siHGF transfection (Figure 1(Bi–iv)), compared to scrambled siRNA-transfected OMFs (Figure 4(Bv–viii)). The percentage of wound closure rates were significantly reduced by siHGF transfection at 24 h (*p* = 0.002), 48 h (*p* = 0.022) and 72 h (*p* = 0.015), to between 20% and 30% of the wound closure rates determined in scrambled siRNA-transfected OMFs, which approached complete wound closure over the 72 h culture period.

### 2.2. Exogenous Transforming Growth Factor-β_1_ (TGF-β_1_) Supplementation Inhibits Oral Mucosal Fibroblast Proliferation and In Vitro Scratch Wound Repopulation

**Figure 2:**
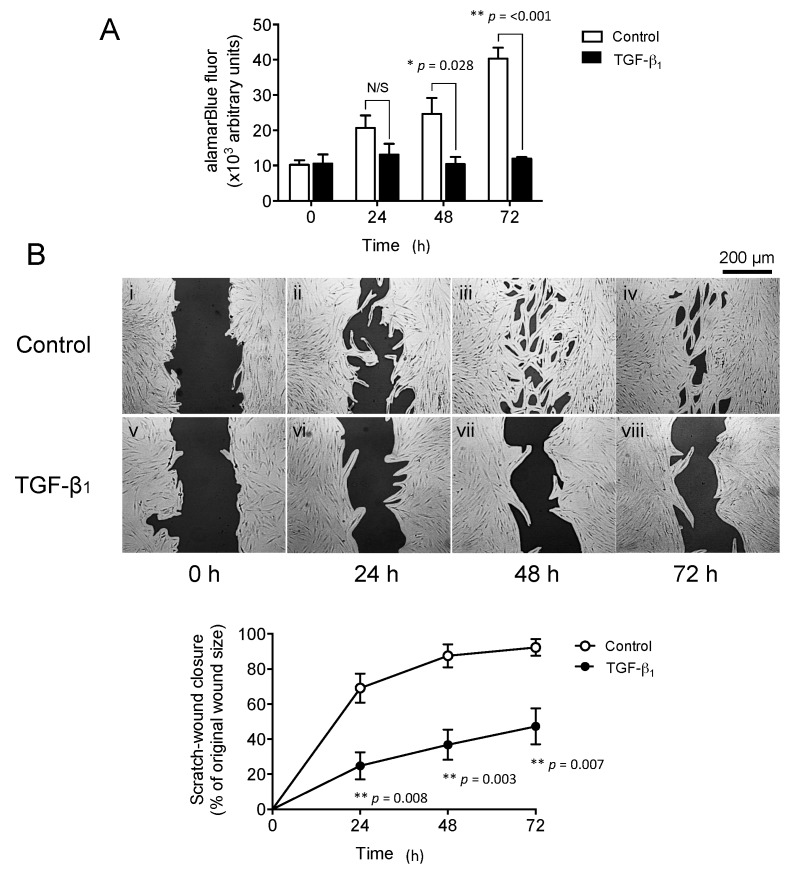
Transforming growth factor-β_1_ (TGF-β_1_) supplementation attenuates OMF cell functions. The effects of TGF-β_1_ (10 ng/mL, solid bars/lines) treatment for up to 72 h on OMF (**A**) proliferation and (**B**) in vitro scratch wound repopulation abilities were assessed. Time-lapse images are representative of 3 independent scratch wound experiments (scale bar = 200 μm, original magnification 100×). Data is shown as mean ± S.E. for *n* = 3 independent experiments (* *p* ≤ 0.05, ** *p* ≤ 0.01, vs. serum-free F-SCM controls; N/S, no significance).

OMF proliferation, with and without exogenous TGF-β_1_ supplementation, over 72 h in culture, is shown in Figure 2A. OMF proliferation was significantly inhibited in TGF-β_1_-supplemented cultures at 48 and 72 h (*p* = 0.028 and *p* < 0.001, respectively). Assessment of OMF by in vitro scratch wound repopulation, with and without exogenous TGF-β_1_ supplementation over 72 h, also demonstrated that OMF wound repopulation/closure responses were significantly impaired by exogenous TGF-β_1_ supplementation (Figure 2(Bi–iv)), compared to untreated OMF controls (Figure 2(Bv–viii)) The rates of wound closure were significantly reduced by TGF-β_1_ supplementation at 24 h (*p* = 0.008), 48 h (*p* = 0.003), and 72 h (*p* = 0.007), to between 30% and 50% of the wound closure rates observed in untreated OMF controls, which exhibited almost complete wound closure over 72 h.

### 2.3. Exogenous TGF-β_1_ Supplementation Suppresses HGF Expression in a Similar Manner to Targeted HGF Knockdown

**Figure 3:**
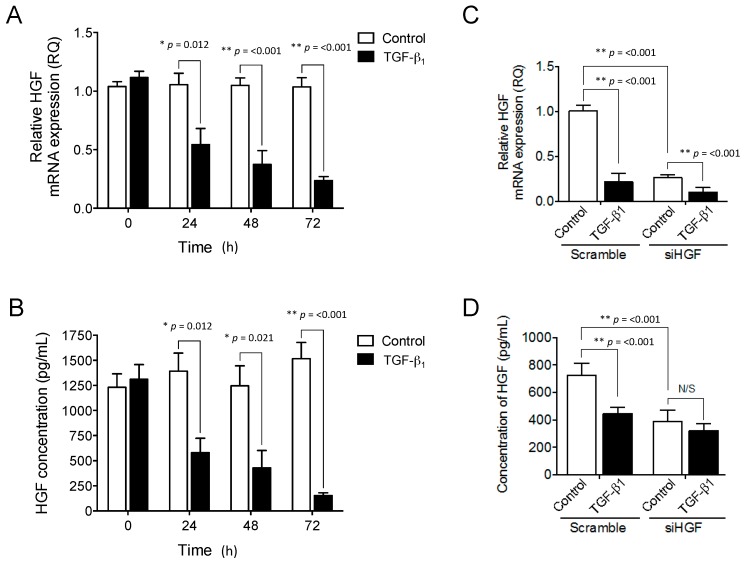
TGF-β_1_ supplementation or HGF knockdown inhibit both HGF mRNA expression and protein synthesis. OMFs were maintained in serum-free F-SCM (open bars) or serum-free F-SCM containing TGF-β_1_ (10 ng/mL, solid bars), for 72 h. OMF (**A**) HGF mRNA expression and (**B**) HGF protein levels were assessed. OMFs were transiently transfected with either siRNA specific for HGF (siHGF) or a scrambled siRNA. siHGF- and scrambled siRNA-transfected were maintained in serum-free F-SCM (open bars) or serum-free F-SCM containing TGF-β_1_ (10 ng/mL, solid bars) for 72 h. The relative extent of HGF expression in siHGF and scrambled control cultures was confirmed by (**C**) RT-qPCR and (**D**) ELISA. RT-qPCR data is shown as mean ± S.E. for *n* = 6 independent experiments, and ELISA data is displayed as mean ± S.E. for *n* = 4 independent experiments (* *p* ≤ 0.05, ** *p* ≤ 0.01, vs. serum-free F-SCM controls; N/S, no significance).

Exogenous TGF-β_1_ supplementation could reduce HGF expression within 3D fibroblast-populated collagen gels [9]. We therefore examined whether the retardation of OMF proliferation, and wound repopulation responses with exogenous TGF-β_1_ supplementation, were a consequence of reduced HGF expression. Exogenous TGF-β_1_ supplementation significantly downregulated (2-fold) HGF mRNA expression by OMFs (Figure 3A) at 24 h (*p* = 0.012), which was maintained over 48 and 72 h in culture (5-fold inhibition by 72 h, both *p* < 0.001). In contrast, untreated OMF controls exhibited consistently high HGF expression levels over 72 h in culture. Following 24 h of TGF-β_1_ treatment, HGF protein levels mirrored the down-regulation observed at the mRNA level (Figure 3B), with significant (approximately 60%) reduction (*p* = 0.012). Similar levels were maintained at 48 h (*p* = 0.021), and a 90% reduction in HGF levels was observed by 72 h (*p* < 0.001). In contrast, untreated OMF controls maintained consistently high levels of HGF over the 72 h culture period. 

We next assessed the influence of transient knockdown of HGF expression by siHGF transfection, on TGF-β_1_ responses in OMFs. RT-qPCR analysis revealed that siHGF significantly down-regulated HGF mRNA expression in both untreated control, and TGF-β_1_-treated, OMFs (4-fold and 2-fold, respectively), compared to scrambled siRNA-transfected OMFs, with and without TGF-β_1_ supplementation (both *p* < 0.001, Figure 3C). Furthermore, HGF mRNA expression in both siHGF- and scrambled siRNA-transfected OMFs was significantly down regulated (4-fold and 2-fold, respectively) by TGF-β_1_ supplementation (both *p* < 0.001, Figure 3A). These findings were further validated by ELISA, confirming HGF protein reduction in both TGF-β_1_-treated OMFs (1.5-fold) and in siHGF-transfected OMFs (2-fold), compared to scrambled siRNA-transfected control OMFs (both *p* < 0.001, Figure 3D). However, no significant reductions in HGF levels were apparent between siHGF-transfected OMFs with or without exogenous TGF-β_1_ supplementation (*p* < 0.05, Figure 3B).

### 2.4. HGF Knockdown Impairs Oral Mucosal Fibroblast Resistance to TGF-β_1_-Driven, Myofibroblast Differentiation

**Figure 4:**
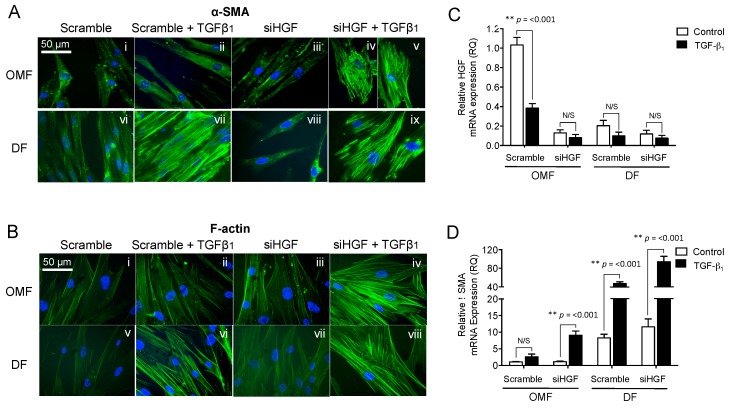
Knockdown of HGF, followed by TGF-β_1_ supplementation, is sufficient to differentiate OMFs into myofibroblasts. siHGF- and scrambled siRNA-transfected OMFs and DFs were growth-arrested and maintained in serum-free F-SCM or serum-free F-SCM containing TGF-β_1_ (10 ng/mL), for 72 h. The effects of exogenous TGF-β_1_ supplementation on (**A**) α-SMA staining and (**B**) F-actin cytoskeletal reorganisation/cellular morphology were assessed. α-SMA and F-actin images are representative of one independent experiment of three (scale bar = 50 μm, original magnification 400×). siHGF- and scrambled siRNA-transfected OMFs, and patient-matched DFs, were further assessed for (**C**) HGF and (**D**) α-SMA mRNA expression. Images are representative of *n* = 3 independent experiments. Graphical data is shown as mean ± S.E. for *n* = 6 independent experiments (* *p* ≤ 0.05, ** *p* ≤ 0.01; N/S, no significance).

Meran et al. demonstrate that OMFs resist TGF-β_1_-driven fibroblast-myofibroblast differentiation [13]. In siHGF- and scrambled siRNA-transfected, OMFs without TGF-β_1_ supplementation had spindle-shaped, fibroblastic morphology, and were negative for α-SMA staining and F-actin remodelling at 72 h in culture (Figure 4(Ai,iii,Bi,iii)). However, TGF-β_1_ supplementation increased the extent of positive staining for α-SMA in siHGF-transfected OMFs, coupled with larger, polygonal morphology, indicative of myofibroblast formation (Figure 4(Aiv–v,Biv)). These OMFs also demonstrated prominent F-actin reorganisation into thick cell-spanning filaments. In contrast, TGF-β_1_ supplementation in scrambled siRNA-transfected OMFs did not induce α-SMA staining, morphological changes or F-actin reorganisation (Figure 4(Aii,Bii)). The α-SMA/F-actin staining and morphological changes evident in siHGF-transfected OMFs resembled typical characteristics of myofibroblast differentiation apparent in patient-matched DF control cultures supplemented with TGF-β_1_ (Figure 4(Avii,ix,Bvi,viii)). Control DF expression of α-SMA/F-actin stress fibres were not apparent in the absence of TGF-β_1_ supplementation, regardless of transfection (Figure 4(Avi,viii,Bv,vii)).

These findings were confirmed by RT-qPCR analysis of HGF and α-SMA mRNA expression (Figure 4C,D respectively). siHGF significantly down-regulated HGF expression in both untreated control and TGF-β_1_-treated OMFs (10-fold; *p* < 0.001 and 4-fold; *p* < 0.001, respectively), when compared to scrambled siRNA-transfected OMFs (Figure 4C). Similarly, HGF expression in the scrambled siRNA-transfected OMFs was significantly downregulated by TGF-β_1_ supplementation (2.5-fold, *p* < 0.001). HGF expression in OMFs under TGF-β_1_ and siHGF conditions was equivalent to mRNA expression observed in DFs.

No significant effects on α-SMA expression in scrambled siRNA-transfected OMFs, with and without TGF-β_1_ supplementation, were observed (*p* > 0.05, Figure 4D). However, siHGF-transfected OMFs demonstrated significant increases in α-SMA expression after TGF-β_1_ supplementation (9-fold induction, *p* < 0.001). The extent of α-SMA expression in siHGF-transfected OMFs treated with TGF-β_1_ was equivalent to mRNA levels observed in DF control cells (Figure 4D); whilst TGF-β_1_ supplementation resulted in significantly larger increases in α-SMA expression (7-fold induction, *p* < 0.001) in scrambled siRNA-transfected DFs, and in siHGF-transfected DFs (9-fold induction, *p* < 0.001). These results suggested that a 2-fold reduction of HGF expression in DF was sufficient to cause a significant increase in α-SMA mRNA (control scramble DF vs. siHGF DF *p* = 0.233; TGF-β_1_-treated DF vs. siHGF DF *p* = 0.004). 

### 2.5. Full-Length HGF and Truncated HGF isoforms (HGF-NK), NK1 and NK2, Expression Levels Differ between Oral and Dermal Fibroblasts and Are Differentially Influenced by TGF-β_1_

**Figure 5:**
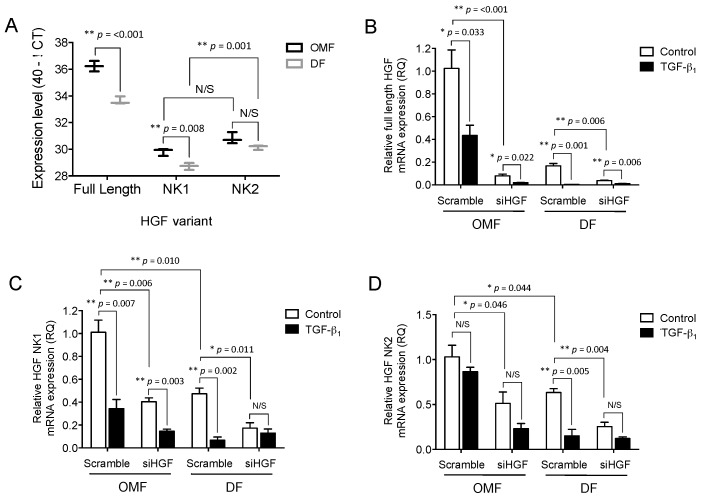
HGF isoforms are differentially expressed in OMFs vs. DFs, and following supplementation with TGF-β_1_. OMFs and DFs were transiently transfected with either siHGF or scrambled siRNA. siHGF- and scrambled siRNA-transfected cells were growth-arrested and maintained in serum-free F-SCM (open bars), or serum-free F-SCM containing TGF-β_1_ (10 ng/mL, solid bars), for 72 h. (**A**) Differences in basal levels of full-length HGF and truncated NK1/NK2 isoform mRNA expression in OMF (black lines) and DF (grey lines) were analysed by RT-qPCR, and are displayed as 40-ΔCT. The expression of (**B**) full-length HGF, (**C**) truncated NK1, and (**D**) truncated NK2 mRNA, was assessed by RT-qPCR. Data is shown as mean ± S.E. for *n* = 3 independent experiments (* *p* ≤ 0.05, ** *p* ≤ 0.01; N/S, no significance).

We next sought to delineate the extent to which full-length HGF, and the truncated NK1/NK2 isoforms, participated in mediating the observed OMF responses. RT-qPCR analysis was used to assess basal expression of full-length, NK1, and NK2 mRNA, and demonstrated that OMFs had greater levels of full-length HGF, compared to the NK1 and NK2 variants, which had equivalent expression levels. In patient-matched DFs, there was significantly less basal expression of full-length (*p* < 0.001), and NK1 (*p* = 0.008) HGF, than in OMFs. However, DF basal expression of NK2 was similar to that of OMFs (by 40^−Δ*C*t^ method of representation; Figure 5A). Furthermore, NK2 mRNA levels in DFs were significantly higher than NK1 (*p* < 0.001, Figure 5A). Full-length HGF was more highly expressed than NK1 or NK2 mRNA in both cell types (*p* < 0.001).

We next assessed the extent to which siHGF transfection and exogenous TGF-β_1_ supplementation down-regulated full-length HGF and the truncated NK1/NK2 isoforms in OMFs and patient-matched DFs. Transfection with siHGF significantly down-regulated full-length HGF (10-fold, *p* < 0.001, Figure 5B) and NK1 (3-fold, *p* = 0.006, Figure 5C), in OMFs; NK2 expression was much less affected (2-fold, *p* = 0.046, Figure 5D). Furthermore, despite siHGF transfection reducing full-length HGF, NK1 and NK2 expression in OMFs to equivalent levels observed in DFs (Figure 5B–D), full-length HGF (Figure 5B) and NK1 (Figure 5C) expression was significantly down-regulated by TGF-β_1_ supplementation in both the siHGF- (4-fold for full-length HGF, 3-fold and NK1, *p* = 0.022 and *p* = 0.003, respectively) and scrambled siRNA-transfected OMFs (2.5-fold for full-length HGF, and 3-fold for NK1, *p* = 0.033 and *p* = 0.007, respectively). No significant differences in NK2 expression were evident in either the siHGF- or scrambled siRNA-transfected OMFs, following TGF-β_1_ supplementation (both *p* > 0.05, Figure 5D).

siHGF also downregulated full-length HGF (4-fold, *p* = 0.004), NK1 (3-fold, *p* = 0.011) and NK2 (2.5-fold, *p* = 0.004) in patient-matched DFs, compared to scrambled siRNA-transfected DFs (Figure 5B–D, respectively). Furthermore, although exogenous TGF-β_1_ supplementation significantly down-regulated full-length HGF expression in both siHGF- (2-fold, *p* = 0.006) and scrambled siRNA-transfected DFs (10-fold, *p* = 0.001, Figure 4B), TGF-β_1_ only downregulated NK1 (6-fold, *p* = 0.002) and NK2 (4-fold, *p* = 0.005) expression in scrambled siRNA-transfected DFs (Figure 4C,D, respectively), with no significant NK1 or NK2 expression changes in siHGF-transfected DFs, following TGF-β_1_ supplementation (both *p* > 0.05).

## 3. Discussion

The preferential wound healing capabilities of OMFs have been well documented [2,3], as has HGF’s facilitation of mitogenic, motogenic, morphogenic, and anti-fibrotic responses [21,22]. In light of the evidence confirming elevated HGF expression and protein levels in OMFs, compared to DFs [18], this study investigated the extent to which HGF plays a role in mediating the enhanced proliferative/migratory properties of OMFs and their resistance to TGF-β_1_-driven myofibroblast differentiation, in addition to reciprocated changes in full-length and NK1/NK2 isoform expression.

OMFs possess enhanced proliferative, migratory, and in vitro scratch wound repopulation capabilities, compared to patient-matched DF counterparts [4,5,6]. In the current study, OMFs exhibited rapid proliferation and scratch wound repopulation/closure, together with induction of HGF transcription and increased protein expression. These responses were significantly retarded by exogenous TGF-β_1_ supplementation or HGF knockdown, corresponding with significant reductions in HGF mRNA expression and protein levels. TGF-β_1_ has previously been shown to have detrimental effects on the proliferative and migratory responses in OMFs and other cell types [10,33]. In addition, TGF-β_1_ could reduce HGF expression by various cells [9,34,35,36,37]. Here, we show that HGF knockdown and TGF-β_1_ stimulation have similar resultant consequences on OMF proliferation, migration, and HGF expression. Furthermore, the siHGF experimental protocols utilised herein successfully down-regulated HGF mRNA and protein expression by approximately 80% and 50%, respectively. Studies have shown that HGF is capable of suppressing elevated TGF-β_1_ cell signalling and downstream events [37,38,39,40,41]. This intricate interplay between growth factors suggests that HGF knockdown could help to drive fibrotic events in OMFs. Indeed, here, we show for the first time that as with proliferative and migratory responses, OMF resistance to TGF-β_1_-driven fibroblast-myofibroblast differentiation was significantly impaired by exogenous TGF-β_1_ supplementation following HGF knockdown. This increased susceptibility was presumably a consequence of reduced HGF protein expression. Furthermore, previous studies have demonstrated that OMFs and oral mucosal wounds have reduced TGF-β_1_ levels and altered TGF-β_1_ signalling, in addition to higher levels of the anti-fibrotic TGF-β_3_ [14,15,42,43]. Therefore, it is conceivable that heightened HGF levels in OMFs are causative of reduced basal levels of TGF-β_1_ in OMFs, contributing to the reduction in overall TGF-β_1_, in oral mucosal wounds.

OMF anti-proliferative responses to TGF-β_1_, and resistance to differentiation, are regulated through differential Smad3 signalling, and by restricted synthesis and assembly of the matrix polysaccharide, hyaluronan, by hyaluronan synthase 1 and 2 (HAS1 and HAS2), in contrast to DFs [13,15]. As OMF proliferation and resistance to differentiation were significantly attenuated by exogenous TGF-β_1_ supplementation and HGF knockdown, it is interesting to speculate whether HGF influences Smad3 signalling and hyaluronan synthesis/localisation, regulating these responses. HGF/c-MET activate many signalling pathways [21,22,28], however, HGF has also been demonstrated to be capable of antagonising pro-fibrotic effects of TGF-β_1_ through inhibition of phospho-Smad2/3 nuclear translocation, in addition to increasing the expression of Smad regulators, such as TGF-β-induced factor homeobox 1 (TGIF), SnoN, and galectin-7 [37,40,44,45,46]. TGF-β receptors can also activate non-Smad pathways, including ERK1/2, PI3K/Akt, and Rho GTPases [47]. Therefore, as no major differences in c-MET receptor expression have previously been reported between OMFs and DFs [6,16,17,18], it is feasible that HGF mediates preferential healing responses in OMFs by modulating Smad and non-Smad signalling pathways. Further investigations into the differences between OMF and DF c-MET phosphorylation, its membrane localisation, co-receptor activity, and downstream signalling events, in response to TGF-β_1_, HGF, and its variants, will provide detailed mechanistic understanding of the extent of c-MET roles in maintaining phenotype. However, as HGF has also been demonstrated to increase HAS1- and HAS2-derived hyaluronan in fibroblasts from non-oral mucosal sources [48,49], a role for HGF in limiting hyaluronan synthesis by OMFs and subsequent TGF-β_1_-driven myofibroblastic differentiation, may be a characteristic specific to OMFs as part of their highly privileged wound healing phenotype.

HGF can exist as a full-length protein, or as alternative spliced variants, including NK1 and NK2 [26,27,28]. We sought to determine basal levels of full-length HGF and truncated NK1/NK2 isoform expression in OMFs and patient-matched DFs, and the impact of siHGF transfection and exogenous TGF-β_1_ supplementation on overall expression. Basal levels of full-length HGF were higher than both truncated NK1/NK2 isoforms in OMFs, with full-length HGF and NK1 being expressed at significantly higher levels in OMFs than DFs. Interestingly, NK2 demonstrated no significant difference in expression between OMFs and DFs. NK2 mRNA levels in DFs were also significantly higher than NK1. These results suggest support for the contrasting wound healing properties of OMFs and DFs (NK1 as essential for cellular proliferation, motility, and survival, whereas NK2 antagonises the mitogenic activity of HGF, pivotal to its anti-fibrotic functions) [27,32,50,51]. Furthermore, despite full-length HGF and the NK2 isoform having similar c-MET receptor affinities, only full-length HGF activates PI3K/Akt signalling responsible for stimulating mitogenic activity [28,32]. Taken together with the changes observed in this report, it appears that full-length HGF and the NK1 isoform were principally responsible for promoting the preferential proliferative, migratory and resistance to TGF-β_1_-driven fibroblast–myofibroblast differentiation properties of OMFs; although, the precise roles that downstream signalling pathways have in mediating these responses requires further investigation.

Interestingly, TGF-β_1_ supplementation attenuated NK2 expression to a lesser degree. Full-length HGF expression was also more susceptible to siHGF/TGF-β_1_ down-regulation in DFs. In line with these findings, TGF-β_1_ has been previously demonstrated to promote full-length HGF mRNA degradation by microRNA-199, while the NK2 isoform mRNA remained unaffected [35,52]. The 3′UTR (untranslated region) of full-length HGF mRNA differs to the NK2 3′UTR [52], consequently, full-length HGF is distinctly regulated at the post-transcriptional level compared to its antagonist, NK2.

As HGF elicits a protective anti-fibrotic effect in various tissues, including lung, heart, liver, and kidney [37,38,40,53,54,55,56], it is conceivable that HGF protein or gene therapy approaches would offer similar beneficial wound healing, and anti-fibrotic outcomes could be facilitated in skin. Indeed, HGF gene/protein delivery has been demonstrated to promote both chronic wound healing and the resolution of various forms of dermal fibrosis [36,57,58,59]. Through further investigation of the distinct roles of HGF isoforms, these outcomes could be substantially improved. Due to the unavailability of recombinant forms of the different HGF isoforms, future investigations will require the use of overexpression vectors for forced expression of full-length, NK1, NK2, or other NK isoforms of HGF in OMFs/DFs. In combination with experiments utilising custom siRNA, the more specific and detailed roles of these HGF isoforms could then be exemplified. 

## 4. Material and Methods

### 4.1. Reagents

Cell culture reagents used were obtained from Thermo Fisher Scientific (Paisley, UK), Sigma (Poole, UK) or BD Bioscience (Oxford, UK), unless otherwise stated. siRNA transfections reagents were purchased from Thermo Fisher Scientific (Paisley, UK). Human recombinant TGF-β_1_ was from R&D Systems (Abingdon, UK). Reverse-transcription polymerase chain reaction (RT-PCR) and real time quantitative PCR (RT-qPCR) reagents were from Thermo Fisher Scientific (Warrington, UK) and New England Biolabs (Hitchin, UK).

### 4.2. Oral Mucosal and Patient-Matched, Dermal Fibroblast Cultures

Patient-matched OMF and DF were established from oral mucosal and normal skin biopsies (6 mm), obtained with Local Research Ethical Committee approval and informed consent, from adults undergoing minor oral surgical operations at the School of Dentistry, Cardiff University, UK. Cultures were isolated as previously described [5], and cultured in DMEM/F-12 Medium, supplemented with 2 mM l-glutamine, 100 U/mL penicillin, 100 μg/mL streptomycin and 10% foetal calf serum (FCS, Biologic Industries, Cumbernauld, UK) (F-SCM). Cultures were maintained at 37 °C, in 5% CO_2_/95% air.

### 4.3. Short Interfering RNA (siRNA) Transfection

siRNA against HGF (ID 112632, Thermo Fisher Scientific, Waltham, MA, USA) was used to knockdown HGF mRNA expression in DFs/OMFs. Lipofectamine 2000, using an optimised and adapted manufacturer’s protocol, was used to transfect siRNA into the cells (Santa Cruz Biotechnology, Dallas, TX, USA). OMFs were seeded into 35 mm wells in F-SCM at a density of 2 × 10^5^ cells/well. Once 60–70% confluent, 3 μL Lipofectamine 2000, 30 nM HGF siRNA (siHGF) or scrambled siRNA (scr) (a scrambled sequence with no homology to the human genome, ID 4390846, Thermo Fisher Scientific) and 200 μL OPTI-MEM, was mixed and incubated for 30 min at room temperature, before the addition of 800 μL OPTI-MEM (1 mL final volume) (transfection solution). Cells were washed with OPTI-MEM and incubated with the transfection solution for 5 h. F-SCM containing 20% FCS (1 mL) was then added to each well and incubated overnight. Media were replaced with fresh F-SCM, and cells incubated for a further 24 h, before growth-arrest in serum-free F-SCM for 24 h. 

### 4.4. TGF-β_1_ Supplementation

Following growth arrest, cultures were treated with serum-free F-SCM alone, or supplemented with TGF-β_1_ (10 ng/mL), for times up to 72 h. 

### 4.5. Proliferation Assay

Cell numbers were assessed by the addition of 10% AlamarBlue^®^ Cell Viability Reagent (Thermo Fisher Scientific), to the medium in each well (10% *v*/*v*), up to 72 h. Cultures were maintained for 1 h before fluorescence was quantified at excitation (540 nm)/emission (590 nm), using a Fluostar Optima Fluorescence Spectrometer (BMG Lab Technologies, Aylesbury, UK). Data was expressed as arbitrary fluorescence units.

### 4.6. In Vitro Scratch Wound Repopulation

OMFs were seeded in 35 mm culture dishes in F-SCM at a density of 2 × 10^5^ cells/well, and maintained until confluent. OMFs were growth-arrested in serum-free F-SCM for 24 h, prior to a single scratch being made with a sterile pipette across each cell layer. Following serum-free F-SCM removal and PBS washes, OMFs were replenished with serum-free F-SCM supplemented with TGF-β_1_ (10 ng/mL) or serum-free F-SCM alone, and incubated for 72 h. Digital images of the denuded areas were captured for times up to 72 h (original magnification 100×), using an Axiovert 133 Microscope (Carl Zeiss, Welwyn Garden City, UK) fitted with a CCD digital camera (Hamamatsu Photonics, Welwyn Garden City, UK) using Openlab 3.0.4 Software (Improvision, Coventry, UK). Wound closure was quantified using ImageJ Software (ImageJ v1.49h, https://imagej.nih.gov/ij/). Data was expressed as % wound closure/original total wound areas at 0 h.

### 4.7. Reverse Transcription PCR (RT-PCR) and Real Time Quantitative PCR (RT-qPCR)

Cells were lysed with TRIzol Reagent Solution (Thermo Fisher Scientific). Total RNA was isolated by manufacturer’s protocols. RNA was quantified (NanoDrop 1000, Labtech International, Lewes, UK) and RT-PCR was performed using High-Capacity cDNA Reverse Transcription Kits (manufacturer’s instructions). RT-PCR (PTC-225 Thermal Cycler, MJ Research, St. Bruno, QC, Canada), at 25 °C for 5 min, 37 °C, 2 h and 85 °C, 5 min. RT-PCR (negative control) replaced sample RNA sterile nuclease-free water (NFW).

RT-qPCR analysis used MicroAmp 96-well plates and Power SYBR Green Gene Expression Assay (both Thermo Fisher Scientific) with primer sequences (Table 1 and reference gene, GAPDH). RNA was replaced with NFW for negative controls. RT-qPCR used a ViiA-7 Real-Time PCR System (Thermo Fisher Scientific), according to manufacturer’s instructions. RT-qPCR was performed at a final volume of 20 μL per sample (4 μL cDNA, 0.6 μL forward and reverse primers, 10 μL Power SYBR Green Master-Mix and 4.8 μL NFW). Relative quantification was calculated using the comparative *C*_t_ method (*C*_t_, cycle threshold; Δ*C*_t_, *C*_t_ of target gene minus *C*_t_ of reference gene (GAPDH)):

Relative quantification (RQ) = 2^−[Δ*C*t(Experimental) − Δ*C*t (mean control)]^

### 4.8. HGF Enzyme-Linked Immunosorbant Assay (ELISA)

HGF expression by OMFs in culture medium was measured by ELISA (Quantikine Human HGF Immunoassay, R&D Systems), according to manufacturer’s instructions. Absorbance at 450 nm was measured by Fluostar Optima Microplate Reader (BMG Lab Technologies) and expressed as pg HGF/mL.

### 4.9. Immunocytochemistry

Immunocytochemistry was used to visualise α-SMA stress fibres and filamentous F-actin. DFs/OMFs were seeded in Nunc 8-well Permanox chamber slides (Thermo Fisher Scientific) in F-SCM at a density of 2.5 × 10^4^ cells/well and maintained at 37 °C in a 5% CO_2_/95% air atmosphere, until 60–70% confluent. Cells were subsequently transfected with siHGF. Scrambled siRNA-transfected OMFs and patient-matched DFs, served as controls. Cells were growth-arrested in serum-free F-SCM for 24 h, before incubation in serum-free F-SCM, with or without TGF-β_1_ (10 ng/mL). Cells were fixed in ice-cold 4% paraformaldehyde in PBS (Santa Cruz Biotechnology, 200 μL), for 10 min at room temperature, washed with PBS (×2), permeabilised with 0.1% *v*/*v* Triton X-100 (Sigma) in PBS (100 μL) for 10 min; and blocked with 1% *w*/*v* bovine serum albumin (Sigma) in PBS (BSA-PBS, 200 μL) at room temperature for 30 min. After washing (×3 with 0.1% *w*/*v* BSA-PBS0 α-SMA stress fibres were stained using monoclonal mouse-anti-human α-SMA primary antibody (1:200, clone 1A4, Sigma) in 0.1% *w*/*v* BSA-PBS, for 24 h at 4 °C. Cells were washed in 0.1% *w*/*v* BSA-PBS (×3), then incubated with goat-anti-mouse IgG-AlexaFluor488-conjugated antibody (1:1000 in 0.1% BSA–PBS, Thermo Fisher Scientific), for 1 h at room temperature. Direct visualisation of F-actin, used, phalloidin-FITC (1:50 in 0.1% *w*/*v* BSA-PBS, Sigma), for 1 h at room temperature. Nuclei were stained with Hoechst 33258 solution (1:2000 in 0.1% *w*/*v* BSA-PBS, Sigma, St. Louis, MO, USA). Slides were mounted with FluorSave (Merck Millipore, Watford, UK) and viewed by fluorescence microscopy (Leitz Dialux 20EB Fluorescent Microscope, Leica Microsystems, Milton Keynes, UK).

### 4.10. Statistical Analysis

Graphical data was expressed as the mean ± standard error (S.E.). Data was analysed using GraphPad Prism v6.00 (GraphPad Software, San Diego, CA, USA). One-way analysis of variance (ANOVA) was used to identify differences across data groups; and unpaired two-tailed Student’s *t*-tests with Bonferroni correction were performed to determine significance between data groups. In experiments with multiple variables, significance was determined through the use of two-way ANOVA with Tukey’s post-test. Significance was considered at * *p* < 0.05 and ** *p* < 0.01.

## 5. Conclusions

The findings from this study suggest the beneficial cellular functions of OMFs, required for their impeccable ability to heal oral mucosal tissue, was primarily attributed to the high levels of HGF synthesised by these cells. Despite TGF-β_1_ stimulation resulting in reduced proliferation and migration of OMFs, they still resisted myofibroblast differentiation. Decreasing HGF production, prior to TGF-β_1_ stimulation, was sufficient to drive OMF differentiation to myofibroblasts, suggesting that fibroblasts that do not synthesise large amounts of HGF are more likely to undergo TGF-β_1_-driven differentiation, and further highlighting the potential for HGF and its isoforms to be utilised in anti-fibrotic studies. In conclusion, we propose that the importance of full-length HGF and the NK1 isoform lies in mediating “enhanced” OMF wound healing properties, and the interplay between TGF-β_1_ and HGF in regulating these responses (anti-fibrotic); whereas preferential expression of HGF-NK2 can allow cells, especially fibroblasts, to readily undergo TGF-β_1_-driven differentiation to myofibroblasts (pro-fibrotic). Such findings further advocate the potential use of HGF isoforms as therapeutic interventions for the treatment of fibrotic or non-healing situations.

## Figures and Tables

**Table 1:** Forward and reverse primer sequences used for RT-qPCR.

Target	Primer Sequence (5′–3′)
HGF	Forward: GGACGCAGCAAGGGAACAGT
	Reverse: CCCGATAGCTGTGTTCGTGTGGT
Full-length HGF	Forward: ACTGCCGAAATCCAGATGGG
	Reverse: TTGGGAGCAGTAGCCAACTC
Truncated HGF (NK1)	Forward: TGCCATGTGGGCCATTCTAT
	Reverse: TAGTTGCATTTGCACGAACAACA
Truncated HGF (NK2)	Forward: ATGGGCTCTCAACTGATGGTG
	Reverse: AGCGAGAGAGGTAGGGATCA
GAPDH	Forward: CCTCTGACTTCAACAGCGACAC
	Reverse: TGTCATACCAGGAAATGAGCTTGA
